# Schulden, Kredite und unbezahlte Rechnungen unter Nutzer*Innen teilstationärer und stationärer psychiatrischer Behandlung in Berlin

**DOI:** 10.1007/s00115-020-01013-9

**Published:** 2020-10-14

**Authors:** Stefanie Schreiter, Sascha Heidrich, Andreas Heinz, Wulf Rössler, Reinhard Michael Krausz, Meryam Schouler-Ocak, Felix Bermpohl, Stefan Gutwinski

**Affiliations:** 1 Department of Psychiatry and Psychotherapy, Charité Campus Mitte, Charité – Universitätsmedizin Berlin, corporate member of Freie Universität Berlin, Humboldt-Universität zu Berlin, and Berlin Institute of Health, Charitéplatz 1, 10117 Berlin, Deutschland; 2 Psychiatrische Universitätsklinik der Charité im St. Hedwig Krankenhaus, Berlin, Deutschland; 3 Klinik für Psychiatrie und Psychotherapie, Psychiatrische Universitätsklinik, Universität Zürich, Zürich, Schweiz; 4 Institute of Mental Health and Department of Psychiatry, University of British Columbia (UBC), Vancouver, Kanada

**Keywords:** Schulden, Wohnungslosigkeit, Armut, Psychiatrie, Psychische Erkrankung, Debts, Homelessness, Mental illness, Poverty, Psychiatry

## Abstract

**Hintergrund:**

Bisherige Studien der Allgemeinbevölkerung weisen auf eine Assoziation zwischen psychischen Erkrankungen und verschiedenen Formen finanzieller Schwierigkeiten wie Verschuldung hin.

**Ziel der Arbeit:**

Untersuchung der finanziellen Belastungen und assoziierter Faktoren bei Patient*Innen in (teil-)stationärer psychiatrischer Behandlung.

**Material und Methoden:**

Insgesamt 488 Teilnehmer*Innen einer querschnittlichen Patientenbefragung mittels eines strukturierten Interviews zu soziodemografischen sowie klinischen Variablen in (teil-)stationärer psychiatrischer Behandlung in der psychiatrischen Universitätsklinik der Charité im St. Hedwig, zuständig für einen spezifischen Versorgungsbereich in Berlin, gaben Auskunft zu finanziellen Belastungen.

**Ergebnisse:**

Insgesamt 269 (55,1 %) Teilnehmer*Innen wiesen Schulden, Kredite oder offene Rechnungen auf. Unter den Teilnehmer*Innen, die Auskunft zur Kredit- oder Schuldenhöhe machten (*n* = 215), wies der größte Teil (47,0 %) Schulden oder Kredite in der Höhe zwischen 1000 und 9999 € auf, gefolgt von 36,3 % mit Schulden/Krediten zwischen 10.000 und 99.999 €. In den Regressionsmodellen hinsichtlich des Vorliegens von Schulden erwiesen sich ein jüngeres Alter und das Vorliegen einer Substanzabhängigkeit als signifikant assoziierte Faktoren. 22,3 % der Befragten wiesen Schulden in Höhe >10.000 € auf und lebten von Sozialleistung, sodass eine Überschuldung angenommen werden könnte.

**Diskussion:**

Finanzielle Belastungen und bestehende Schulden sollten im psychiatrischen Bereich stärker in der Praxis erfragt und beachtet werden. Geeignete Unterstützungsformen sollten entwickelt und evaluiert werden.

**Zusatzmaterial online:**

Die Onlineversion dieses Beitrags (10.1007/s00115-020-01013-9) enthält weitere Infomaterialien. Beitrag und Zusatzmaterial stehen Ihnen auf www.springermedizin.de zur Verfügung. Bitte geben Sie dort den Beitragstitel in die Suche ein, das Zusatzmaterial finden Sie beim Beitrag unter „Ergänzende Inhalte“.

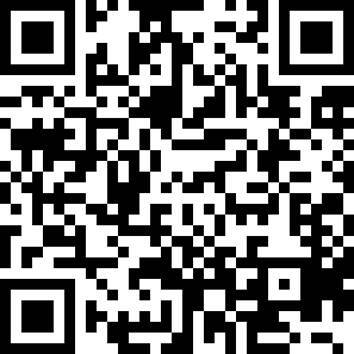

## Abstract (en)

**Background:**

Previous studies among the general population indicated an association between mental illnesses and different forms of financial difficulties, such as indebtedness.

**Objective:**

Investigation of the financial burden and associated factors among inpatients and day clinic patients in psychiatric care.

**Material and methods:**

A total of 488 patients in psychiatric care in the catchment area of the Psychiatric University Hospital Charité at St. Hedwig Hospital participated in a cross-sectional patient survey carried out with a structured interview regarding financial burden, sociodemographic and clinical variables.

**Results:**

Of the participants 269 (55.1%) showed outstanding debts, loans or unpaid bills. Among the participants who were willing to give information about the amount of debts, the majority (47.0%) had debts between 1000 € and 9999 € and 36.3% between 10,000 € and 99,999 €. In the binary regression models, younger age and substance use disorders were factors significantly associated with outstanding debts. Of the participants 22.3% had outstanding debts >10,000 € and were depending on social welfare, so that indebtedness could be assumed.

**Conclusion:**

Financial burdens and outstanding debts among patients in psychiatric care should be inquired about and considered more intensively in practice. Suitable models of support need to be developed and evaluated.

## Hintergrund und Fragestellung

In Deutschland wird aktuell eine Überschuldungsquote von 10 % angenommen, sodass schätzungsweise über 6,9 Mio. Menschen über 18 Jahre in Deutschland bestehende Schulden nicht begleichen können, da kein Vermögen und keine zukünftigen Einnahme absehbar sind [18]. Die Altersstruktur der überschuldeten Personen zeigt, dass die Gruppe der 30- bis 39-Jährigen mit jeder 5. Person die höchste Verschuldungsquote aufweist [18]. Wie das Statistische Bundesamt in einer Untersuchung von 559 der insgesamt rund 1450 Schuldnerberatungsstellen in Deutschland feststellte, lagen *Erkrankungen, Sucht* oder *Unfälle* mit 15,8 % nach der *Arbeitslosigkeit* auf Rang 2 der Hauptursachen für eine Überschuldung [19]. Laut den Ergebnissen einer Metaanalyse ist die Odds Ratio bezüglich des Vorliegens einer psychischen Erkrankung unter verschuldeten Personen gegenüber Personen ohne Schulden 3‑fach erhöht [16]. Bisherige Studien zeigten vor allem Zusammenhänge zwischen Überschuldung und seelischen Störungen („common mental disorders“) [13–16] und globaler psychischer Belastung (ermittelt mit dem General Health Questionnaire; [7, 16]) sowie Drogengebrauch, „problem drinking“ und Rauchen [9, 13, 16]. Die eindeutigste Datenlage zu psychiatrischen Diagnosen findet sich bei depressiven Störungen und zeigt kontrolliert für verschiedene konfundierende Faktoren und in standardisierten Erhebungen einen signifikanten Zusammenhang zum Faktor Schulden [13, 15, 16]. Ähnliche Befunde zeigen sich in einzelnen Studien auch bei Angsterkrankungen [4, 13] und psychotischen Störungen [9]. Zudem ist das Suizidrisiko im Zusammenhang mit Schulden erhöht: Die Rate von Suizidversuchen ist bei überschuldeten Personen 5‑mal so hoch wie in der Allgemeinbevölkerung [7], bei Personen mit einem durchgeführten Suizid ist das Risiko für das Vorliegen von Schulden 8‑fach erhöht [16].

Hinsichtlich des Zusammenhangs zwischen objektiven Variablen der Überschuldung und dem Vorliegen einer psychischen Erkrankung fanden einige Studien Zusammenhänge zwischen der objektiven Höhe und Anzahl der Schulden und der Schwere der Gesundheitsprobleme [9, 13, 16]. Allerdings bleiben die Mechanismen des Zusammenwirkens zwischen Schulden und Gesundheitsproblemen bzw. psychischer Erkrankung ungeklärt, wobei Studien auf verschiedene psychologische Aspekte der Verarbeitung hindeuten, wie beispielsweise (mal)adaptive Copingstrategien [8], Sorgen und Stress bezüglich Schulden [4] oder Gefühle von Hoffnungslosigkeit [12].

Bisherige Studien untersuchten den Zusammenhang zwischen psychischen Erkrankungen und Verschuldung in Bevölkerungsstichproben und spezifischen Populationen wie Studenten, ältere Menschen oder Klienten von Schuldenberatungen [16]. Untersuchungen unter psychiatrischen Patient*Innen sind uns bisher aus Deutschland nicht bekannt. Angesichts der Erreichbarkeit möglicher Angebote für Menschen mit psychischen Erkrankungen und finanzieller Belastung erscheint die nähere Betrachtung des Vorliegens von Verschuldung in einer Stichprobe von bereits bestehenden Nutzer*Innen des Gesundheitssystems sinnvoll. Wir führten daher eine explorative querschnittliche Untersuchung hinsichtlich des Vorliegens finanzieller Belastungen und assoziierter soziodemografischer wie klinischer Faktoren unter Patient*Innen im Rahmen einer (teil-)stationären psychiatrischen Behandlung in Berlin durch.

## Studiendesign und Untersuchungsmethoden

### Durchführung und Stichprobe

Zwischen dem 15.03. und dem 15.09.2016 führten wir eine Befragung in Form eines strukturierten Interviews zu Schulden und anderen soziodemografischen und krankheitsbezogenen Variablen unter allen Patient*Innen der Psychiatrischen Universitätsklinik der Charité im St. Hedwig Krankenhaus durch, die sich in (teil-)stationärer psychiatrischer Behandlung befanden.

Eine ausführliche Beschreibung des Studiendesigns wurde an anderer Stelle veröffentlicht [17].

Die Ethikkommission der Charité – Universitätsmedizin Berlin bewertete die Studie positiv (Nummer: EA1/291/15). Eine schriftliche Einwilligung vor der Studienteilnahme wurde eingeholt. Die Befragung dauerte durchschnittlich eine Stunde; 11 Interviews (2 %) erfolgten mithilfe eines professionellen Sprach- und Kulturvermittlers.

Von den 1251 im Studienzeitraum zu einer (teil-)stationären psychiatrischen Behandlung aufgenommenen Personen nahmen 540 (43,2 %) an der Befragung teil. 328 (26,2 %) lehnten eine Teilnahme ab und 383 (30,6 %) konnten zur Befragung nicht erreicht werden (siehe [17]). 488 Teilnehmer*Innen gaben Auskunft zu bestehenden Schulden, Krediten und offenen Rechnungen. Nach einem Gruppenvergleich zwischen Personen mit und ohne Schulden erfolgte zum Ausschluss von Personen mit geringen Schulden eine Subgruppenanalyse von Personen mit Schulden >1000 € vs. keine Schulden.

### Messinstrumente

Soziodemografische Variablen, das Vorliegen von Schulden und die Schuldenhöhe sowie das Nutzungsverhalten innerhalb des Versorgungssystems und Variablen zur Verschuldungssituation wurden im Rahmen des strukturierten Interviews erfasst. Bei fehlenden oder unklaren Daten wurden diese mit der Dokumentation der Sozialarbeiter*Innen der Klinik abgeglichen. Psychiatrische Diagnosen basierten auf den Entlassungsdiagnosen nach den Kriterien des ICD-10 [6].

Die Erfassung der Wohnsituation basierte auf der Hauptwohnform in den letzten 30 Tagen vor der Klinikaufnahme.

### Statistische Analysen

Statistische Analysen erfolgten mittels SPSS 19 [20]. Deskriptive Analysen erfolgten mit den entsprechenden statistischen Parametern (Mittelwert, Standardabweichung, Median, Quartile). Gruppenvergleiche erfolgten entsprechend den vorliegenden Daten entweder mittels χ^2^- bzw. T‑Test; im Falle nicht normalverteilter Daten wurde der Mann-Whitney-Test durchgeführt. Signifikante Faktoren der Gruppenvergleiche wurden als Faktoren in ein binär logistisches Regressionsmodell eingeführt, wobei im 1. Modell das Vorliegen von Schulden, Krediten oder offenen Rechnungen und im 2. Modell das Vorliegen von Schulden über 1000 € als abhängige Variable diente. Es erfolgte eine Anpassung des *p*-Werts mittels Bonferroni-Methode.

## Ergebnisse

Von den 488 Teilnehmer*Innen, die Auskunft über ihre finanzielle Situation gaben, wiesen 269 (55,1 %) Schulden, Kredite oder offene Rechnungen auf. Unter den Teilnehmer*Innen, die Auskunft zur Kredit- oder Schuldenhöhe machten (*n* = 215), wiesen 14,4 % Schulden oder Kredite in der Höhe von unter 1000 € auf, 47,0 % zwischen 1000 und 9999 €, 36,3 % zwischen 10.000 und 99.999 € und 2,3 % über 100.000 € (Tab. 1).

**Table 1:** 

Schuldenhöhe (*n* = 215)	Anzahl Patient*Innen
<1000 €	31 (14,4 %)
1000–9999 €	101 (47,0 %)
10.000–99.999 €	78 (36,3 %)
>100.000 €	5 (2,3 %)

Hinsichtlich soziodemografischer und klinischer Faktoren waren Teilnehmer*Innen mit Schulden, Krediten oder offenen Rechnungen signifikant häufiger jüngeren Alters, männlichen Geschlechts und in Wohnungslosigkeit; sie lebten signifikant häufiger von Sozialleistungen, wiesen signifikant häufiger mehrere psychiatrische Komorbiditäten auf und waren signifikant häufiger an folgenden psychischen Störungen erkrankt: organische psychische Störungen, schädlicher Gebrauch einer Substanz und Substanzabhängigkeiten sowie Persönlichkeitsstörungen. Sie wiesen signifikant seltener affektive Störungen und Intelligenzminderungen auf (Tab. 2). Im Vergleich zwischen Teilnehmer*Innen ohne Schulden oder Krediten und Teilnehmer*Innen mit Schulden oder Krediten über 1000 € wiesen Teilnehmer*Innen mit Schulden oder Krediten über 1000 € neben den zuvor beschriebenen Gruppenunterschieden zusätzlich signifikant seltener psychotische Erkrankungen auf (s. Zusatzmaterial online eTabelle 1).

**Table 2:** 

Schulden, Kredite oder unbezahlte Rechnungen (*n* = 488)^a^	*Vorliegende *Schulden, Kredite oder unbezahlte Rechnungen	*Keine* Schulden, Kredite oder unbezahlte Rechnungen	Statistik
Anzahl Teilnehmer*Innen	269 (55,1 %)	219 (44,9 %)	–
*Anzahl männliche Teilnehmer*^b^	175 (65,1 %)	109 (50,0 %)	**X**^**2**^**(1)** **=** **11,22; *p*** **=** **0,001**
*Alter (M* *±* *SD)*	39,89 (±11,84)	44,51 (±17,05)	**T** **=** **3,40; *p*** **=** **0,001**
*Bildungsjahre (Median [IQR])*	13,5 (11,5–16,0)	15,0 (12–0–17,0)	Z = −1,89; *p* = 0,059
*Wohnstatus*^c^			**X**^**2**^**(3)** **=** **10,69; *p*** **=** **0,014**
Eigene Wohnung	152 (57,4 %)	142 (65,4 %)	–
Gesundheitsbezogene Einrichtungen	45 (17,0 %)	36 (16,5 %)	–
Wohnungslos	42 (15,8 %)	14 (6,5 %)	–
Bei Freunden/Familie	26 (9,8 %)	25 (11,5 %)	–
*Einkommen*			**X**^**2**^**(2)** **=** **24,64; *p*** **<** **0,001**
Gehalt (Voll- oder Teilzeitarbeit, Ausbildung, BAföG-Bezug, Erspartes)	46 (18,5 %)	49 (24,6 %)	–
Sozialleistungen	196 (78,7 %)	122 (61,3 %)	–
Altersrente	7 (2,8 %)	28 (14,1 %)	–
*Verheiratet oder in fester Partnerschaft*	76 (28,5 %)	57 (26,3 %)	X^2^(1) = 0,29; *p* = 0,332
*Im Ausland geboren*	65 (24,3 %)	45 (20,6 %)	X^2^(1) = 0,90; *p* = 0,210
*Psychische Erkrankungen*			
Organische psychische Störungen	8 (3,0 %)	16 (7,3 %)	**X**^**2**^**(1)** **=** **4,85; *p*** **=** **0,023**
Psychotische Erkrankungen	60 (22,3 %)	62 (28,3 %)	X^2^(1) = 2,32; *p* = 0,078
Substanzabhängigkeit (außer Nikotin)	160 (59,5 %)	64 (29,2 %)	**X**^**2**^**(1)** **=** **44,50; *p*** **<** **0,001**
Schädlicher Gebrauch einer Substanz (außer Nikotin)	63 (23,4 %)	32 (14,6 %)	**X**^**2**^**(1)** **=** **5,97; *p*** **=** **0,009**
Affektive Störungen	72 (26,8 %)	87 (39,7 %)	**X**^**2**^**(1)** **=** **9,23; *p*** **=** **0,002**
Angststörungen	10 (3,7 %)	9 (4,1 %)	X^2^(1) = 0,05; *p* = 0,502
Persönlichkeitsstörungen	64 (23,8 %)	35 (16,0 %)	**X**^**2**^**(1)** **=** **4,55; *p*** **=** **0,021**
Intelligenzminderungen	3 (1,1 %)	9 (4,1 %)	**X**^**2**^**(1)** **=** **4,51; *p*** **=** **0,033**
*Anzahl psychiatrischer Diagnosen nach ICD-10 (außer Nikotin) (Median [IQR])*	1,6 (1–2)	1,4 (1–2)	**Z** **=** **−2,68; *p*** **=** **0,007**
*Alter der ersten psychiatrischen Behandlung (Median [IQR])*	26,0 (±12,57)	26,5 (±15,90)	Z = −0,66; *p* = 0,512
Anzahl der Teilnehmer*Innen mit einem Suizidversuch in der Vorgeschichte	91 (33,8 %)	60 (27,4 %)	X^2^(1) = 2,34; *p* = 0,076
*Vorliegen einer gesetzlichen Betreuung*	62 (23,1 %)	49 (22,5 %)	X^2^(1) = 0,03; *p* = 0,476

^a^ *n* = 52 fehlend oder ausgeschlossen

^b^ Ein Teilnehmer mit dem Geschlecht Transgender wurde bei Geschlecht nicht berücksichtigt

^c^ Der Wohnstatus wurde in vier Gruppen eingeteilt: wohnungslose Teilnehmer*Innen (einschließlich Menschen, die direkt auf der Straße oder in sonstigen Verschlägen leben, in Notunterkünften oder sonstigen Einrichtungen der Wohnungslosenhilfe unterkommen, in Flüchtlingsheimen oder Frauenhäusern); Teilnehmer*Innen in einer eigenen Wohnung oder Wohneigentum; Teilnehmer*Innen in Einrichtungen des Gesundheitswesens bzw. der Eingliederungshilfe (therapeutische Wohngemeinschaften, Trägerwohnungen des betreuten Einzelwohnens, Übergangswohnheime, Krankenheime etc.); Teilnehmer*Innen, die bei Freunden, Bekannten oder Familie lebten

*IQR* Interquartilsabstand

Nach Einführung signifikanter Variablen der Gruppenvergleiche in ein binär logistisches Regressionsmodell zur Vorhersage des Vorliegens von Schulden, Krediten oder offenen Rechnungen ergaben sich ein jüngeres Alter (OR = 0,98; Bonferroni-korrigiertes *p* = 0,036) und das Vorliegen einer Substanzabhängigkeit (OR = 2,41; Bonferroni-korrigiertes *p* < 0,001) als signifikant assoziierte Faktoren (Tab. 3). Im 2. Modell zur Vorhersage des Vorliegens von Schulden oder Krediten über 1000 € ergaben sich ebenfalls ein jüngeres Alter (OR = 0,98; Bonferroni-korrigiertes *p* = 0,018) und das Vorliegen einer Substanzabhängigkeit (OR = 2,41; Bonferroni-korrigiertes *p* = 0,040) als signifikant assoziierte Faktoren (Tab. 3).

**Table 3:** 

	Schulden, Kredite oder unbezahlte Rechnungen (*n* = 441)		Schulden >1000 €	
Variablen	Adjustierte OR (95 %-KI)	p*	Adjustierte OR (95 %-KI)	p**
*Geschlecht (männlich vs. weiblich)*	0,63 (0,41–0,98)	0,084	0,69 (0,42–1,12)	0,260
*Alter*	0,98 (0,96–1,00)	0,036	0,97 (0,96–0,99)	0,018
*Wohnstatus*^*a*^				
Eigene Wohnung	1	–	1	–
Wohnungslos	1,81 (0,86–3,80)	0,232	1,67 (0,78–3,56)	0,372
Gesundheitsbezogene Einrichtungen	0,83 (0,46–1,49)	1	1,05 (0,56–1,99)	1
Bei Freunden/Familie	0,86 (0,42–1,77)	1	0,95 (0,44–2,06)	1
*Einkommen (Gehalt/Altersrente vs. Sozialleistungen)*	0,66 (0,41–1,08)	0,198	0,60 (0,34–1,04)	0,132
*Organische psychische Störungen*	0,76 (0,27–2,20)	1	0,45 (0,12–1,71)	0,486
*Psychotische Erkrankungen*	–	–	0,64 (0,29–1,38)	0,506
*Schädlicher Gebrauch einer Substanz (außer Nikotin)*	1,02 (0,57–1,82)	1	1,02 (0,54–1,94)	1
*Substanzabhängigkeit (außer Nikotin)*	2,41 (1,48–3,92)	<0,001	2,22 (1,14–4,34)	0,040
*Affektive Störungen*	0,89 (0,51–1,55)	1	0,88 (0,40–1,94)	1
*Persönlichkeitsstörungen*	1,07 (0,55–2,08)	1	0,87 (0,38–2,04)	1
*Intelligenzminderungen*	0,24 (0,05–1,09)	0,128	0,17 (0,03–1,04)	0,112
*Anzahl an psychiatrischen Diagnosen*	1,43 (0,90–2,27)	0,264	1,54 (0,87–2,72)	0,278

Der adjustierte *p*-Wert wurde mittels Bonferroni-Methode berechnet (*p* × 2 für die soziodemografischen und klinischen Faktoren)

^a^Im Vergleich mit eigener Wohnung; der Wohnstatus wurde in vier Gruppen eingeteilt: wohnungslose Teilnehmer*Innen (einschließlich Menschen, die direkt auf der Straße oder in sonstigen Verschlägen leben, in Notunterkünften oder sonstigen Einrichtungen der Wohnungslosenhilfe unterkommen, in Flüchtlingsheimen oder Frauenhäusern); Teilnehmer*Innen in einer eigenen Wohnung oder Wohneigentum; Teilnehmer*Innen in Einrichtungen des Gesundheitswesens bzw. der Eingliederungshilfe (therapeutische Wohngemeinschaften, Trägerwohnungen des betreuten Einzelwohnens, Übergangswohnheime, Krankenheime etc.); Teilnehmer*Innen, die bei Freunden, Bekannten oder Familie lebten

*Signifikanz des Modells: *p* < 0,001

** Signifikanz des Modells: *p* < 0,001

Von der gesamten Stichprobe lebten 71,0 % von Sozialleistungen (*n* = 448); Teilnehmer*Innen mit Schulden, Krediten oder offenen Rechnungen lebten zu 78,7 % von Sozialleistungen (*n* = 448); bei einer Höhe von über 10.000 € (*n* = 60) waren es 80,0 %.

## Diskussion

Dies ist die erste Erhebung finanzieller Belastungen unter Nutzer*Innen des psychiatrischen Gesundheitsversorgungssystems in Deutschland. In unserer Studie wiesen mehr als die Hälfte der Patient*Innen (55,1 %) Schulden, Kredite oder offene Rechnungen auf. Die Mehrzahl (47,0 %) zwischen 1000 und 9999 € gefolgt von 36,3 % mit Schulden, Krediten oder offenen Rechnungen in Höhe von 10.000 bis 99.999 €. Da 80,0 % der Personen mit Schulden, Krediten oder offenen Rechnungen in einer Summe von über 10.000 € von Sozialleistungen leben, ist mindestens hier das Vorliegen einer Überschuldung sehr wahrscheinlich, wobei die Bewertung einer Überschuldung immer eine Einzelfallentscheidung ist und auch von anderen Faktoren abhängt (Alter, Zahl der Gläubiger, finanzielle Situation anderer Haushaltsmitglieder, mögliches Einkommen etc.; [5]). Zukünftige Erhebungen sollten daher interindividuelle Unterschiede insbesondere in Bezug auf das Haushaltseinkommen stärker berücksichtigen.

Davon ausgehend würde es bedeuten, dass eine Überschuldung 22,3 % der Befragten, die Angaben zur Schuldenhöhe machten, betrifft. Damit läge die Zahl deutlich höher als die Überschuldungsrate der deutschen Allgemeinbevölkerung von 10 % [18] und würde somit bisherige Studienergebnisse bestätigen, die eine deutliche Assoziation zwischen psychischen Erkrankungen und Verschuldung belegen [16]. Auch in Bezug auf die Wohnsituation zeigt sich, dass der Faktor Wohnungslosigkeit mit dem Vorkommen von Schulden verbunden ist (75,0 % aller wohnungslosen Teilnehmer wiesen Schulden auf, vgl. eigene Wohnung: 51,7 %). Eine kürzlich veröffentlichte longitudinale Studie weist auf einen Zusammenhang zwischen psychischer Gesundheit und Sorgen um die Bezahlbarkeit von Wohnraum („housing affordability stress“) hin [3]. Zukünftige Erhebungen sollten daher interindividuelle Unterschiede insbesondere in Bezug auf diese Faktoren, wie beispielsweise das Haushaltseinkommen, stärker berücksichtigen.

Die Ergebnisse verdeutlichen die Notwendigkeit einer holistischen Blickweise auf psychische Erkrankungen und soziale Stressoren wie Verschuldung und Armut sowie die Notwendigkeit der Evaluierung und Implementierung geeigneter sozialer Unterstützungen im Gesundheitsbereich psychischer Erkrankungen bzw. die Stärkung der Rolle der sozialen Arbeit. Beispiele hierfür sind in Großbritannien zu finden, wo die Regierung während der letzten Rezession zusätzliche Therapien für Menschen unter ökonomischer Belastung finanzierte und Schuldenberatung in Gesundheitseinrichtungen unterstützte [10].

Ergebnisse der Regressionsmodelle wiesen dabei vor allem auf Substanzabhängigkeiten und ein jüngeres Alter als prädiktive Faktoren für das Vorliegen von Schulden hin. Eine Implementierung geeigneter Unterstützungsformen vor allem im Suchthilfebereich erscheint daher sinnvoll. Umgekehrt stellt sich Frage, ob Angebote zur Beratung hinsichtlich psychischer Erkrankungen und insbesondere Substanzgebrauch z. B. in Einrichtungen wie Schuldnerberatungen implementiert und evaluiert werden sollten. Das jüngere Alter deckt sich mit Erhebungen der Allgemeinbevölkerung, in der ebenfalls hinsichtlich der Altersstruktur eher Menschen jüngeren Alters von Überschuldung betroffen sind [18]. Insbesondere die frühe Unterstützung junger Menschen mit psychischen Erkrankungen hinsichtlich finanzieller Belastungen scheint daher besonders wichtig, um einer Marginalisierung vorzubeugen.

Ein mögliches Instrument zur Prävention von Schulden und Unterstützung finanzieller Belange stellt in Deutschland die gesetzliche Betreuung dar. Allerdings fanden sich keine signifikanten Gruppenunterschiede zwischen Teilnehmer*Innen mit und ohne Schulden, offenen Rechnungen oder Krediten hinsichtlich des Vorliegens einer gesetzlichen Betreuung. Inwieweit jedoch möglicherweise insbesondere eine gesetzliche Betreuung in Fällen bereits bestehender finanzieller Schwierigkeiten eingesetzt wurde, wurde im Rahmen dieser Studie nicht untersucht.

Bezüglich möglicher Ursachen der Überschuldung zeigte sich in einer anderen Querschnittserhebung unter überschuldeten Personen in Schweden, dass überschuldete Teilnehmer*Innen mit einer psychischen Erkrankung jünger waren und häufiger maladaptive Copingstrategien nutzten [8]. Dies wiederum spricht für die Möglichkeit, durch psychotherapeutische Interventionen Einfluss auf das subjektive Erleben finanzieller Belastung zu nehmen, was z. B. in Projekten in Großbritannien durch die Schaffung von Therapien für Menschen unter finanzieller Belastung und Schuldenberatung in Gesundheitseinrichtungen aufgegriffen wurde [10].

An dieser Stelle soll auch auf den Aspekt der finanziellen Teilhabe und Verfügung über eigene finanzielle Mittel hingewiesen werden. In Anbetracht der kürzlichen Einführung des Bundesteilhabegesetzes, das wiederum eine Trennung von beispielsweise Wohn- und anderen Unterstützungsleistungen vorsieht und somit auch mehr Verantwortung und Herausforderung hinsichtlich der Finanzierung der verschiedenen Leistungsträger seitens der Nutzer*Innen birgt, sollten finanzielle Schwierigkeiten stärker berücksichtigt werden [11]. Dabei sollten Schulden sowohl als mögliche Folgen einer psychischen Erkrankung in Betracht gezogen werden sowie auch als Risikofaktor für das Entstehen psychischer Störungen, z. B. von Suchterkrankungen.

### Limitationen

Die Angabe der Schuldenhöhe wurde nicht, z. B. anhand von Kontoauszügen oder Angehörigen überprüft, sodass diese allein auf Aussagen der Teilnehmer*innen beruhen und möglicherweise nicht präzise sind. Allerdings wurden unvollständige oder unklare Angaben mit Dokumentationen der Sozialarbeiter*Innen der Klinik abgeglichen. Zudem ist denkbar, dass Patient*Innen mit Schulden bspw. aus Gründen von Scham eine Teilnahme ablehnten.

Weitere longitudinale Studien – möglichst interventionellen Charakters – wären notwendig, um die Planung der Versorgung effektiver zu gestalten. Die Generalisierbarkeit der Ergebnisse ist eingeschränkt, da es sich um Nutzer*Innen eines spezifischen Behandlungssettings eines Bezirks (Wedding, Moabit, Tiergarten) in Berlin handelt, zudem gibt es ein Stichprobenbias, da in der Studie u. a. signifikant häufiger Personen mit Abhängigkeitserkrankungen, männlichen Geschlechts und jüngeren Alters teilgenommen hatten (siehe Beschreibung der Population in Schreiter et al. [17]). Berlin wies 2019 eine verhältnismäßig hohe Arbeitslosenquote mit 11,4 % auf, allerdings ist diese vergleichbar mit Quoten anderer deutscher Großstädte wie Gelsenkirchen (14,8 %), Duisburg (14,3 %), Essen (14,6 %), Magdeburg (9,9 %) und anderen [21]. Trotzdem ist die Generalisierbarkeit der Ergebnisse eingeschränkt, da der Versorgungsbezirk der hiesigen Klinik (Großbezirk Mitte) im regionalen Sozialbericht Berlin und Brandenburg 2017 mit 24,8 % die zweithöchste Armutsgefährdungsquote der Berliner Bezirke aufweist [1]. Zudem wies der Bezirk Mitte 2018 mit 33,6 % einen vergleichsweise hohen Anteil ausländischer Personen (vgl. Gesamtberlin: 19,5 %) auf [2]. Entsprechend des Sozialberichts haben ausländische Berliner*Innen das höchste Armutsrisiko (28,7 %; [1]). In der hiesigen Untersuchung ergab sich kein signifikanter Unterschied im Vorliegen von Schulden, Krediten oder offenen Rechnung bei im Ausland geborener Teilnehmer*Innen; Gespräche erfolgten mit Sprach- und Kulturmittlern und ausländische Teilnehmer machten 27,3 % der Teilnehmer*Innen aus.

## Schlussfolgerung und Fazit für die Praxis

Mehr als die Hälfte (55,1 %) von Nutzer*Innen eines (teil-)stationären psychiatrischen Behandlungssettings weist Schulden, Kredite und offene Rechnungen auf, die Mehrzahl in einer Höhe von über 1000 €. Unsere Ergebnisse legen nahe, dass mindestens 22,3 % aller Befragten, die Auskunft zur Schuldenhöhe machten, eine Überschuldung aufweisen; dabei liegt die Quote deutlich höher als in der deutschen Allgemeinbevölkerung mit etwa 10 %.

Die regelmäßige Erfassung finanzieller Belastungen oder Schwierigkeiten in der Handhabung finanzieller Belange scheint besonders bedeutsam bei jungen Personen und Menschen mit Abhängigkeitserkrankungen.

Geeignete Unterstützungsformen sollten evaluiert werden. Gleichwohl sollten Unterstützungsmöglichkeiten hinsichtlich psychischer Erkrankungen, insbesondere eines Substanzgebrauchs, auch an Stellen sozialer Unterstützungseinrichtungen angeboten werden.

## Caption Electronic Supplementary Material




