# Supplementary material for: Debts, loans and unpaid bills among day patients and inpatients in psychiatric care in Berlin, Germany
Source: Nervenarzt. 2020 Oct 14;92(11):1172–8. [Article in German] doi: 10.1007/s00115-020-01013-9 (PMC8563525; doi:10.1007/s00115-020-01013-9)
Supplement: Supplementary file 1 [file 115_2020_1013_MOESM1_ESM.docx]

**eTabelle 1:** Subgruppenanalyse: Soziodemografische und klinische Gruppenunterschiede zwischen Teilnehmer*Innen ohne Schulden und Kredite über 1000 € (ausgeschlossen Teilnehmer*Innen mit Schulden und Krediten zwischen 1-1.000 €)

| N=412* | Keine Schulden | Schulden oder Kredite > 1.000 Euro | Statistik |
| --- | --- | --- | --- |
| Anzahl Teilnehmer*Innen | 229 (55,6%) | 183 (44,4%) |  |
| Anzahl männliche Teilnehmer** | 116 (50,9%) | 120 (65,6%) | **X²(1)=8,97; p=0,002** |
| Alter (M±SD) | 44,25 (±16,80) | 39,73 (±10,84) | **T=3,30; p=0,001** |
| Bildungsjahre (Median (IQR)) | 14,0 (12,0-17,0) | 13,5 (11,6-16,0) | Z=-1,59; p=0,112 |
| Wohnstatus*** |  |  | **X²(3)=8,52; p=0,036** |
| Eigene Wohnung | 147 (64,8%) | 101 (56,1%) |  |
| Gesundheitsbezogene Einrichtungen | 37 (16,3%) | 33 (18,3%) |  |
| Wohnungslos | 16 (7,0%) | 28 (15,6%) |  |
| Bei Freunden/Familie | 27 (11,9%) | 18 (10,0%) |  |
| Einkommen |  |  | **X²(2)=23,50; p<0,000** |
| Gehalt (Voll- oder Teilzeitarbeit, Ausbildung, BAföG Bezug, Erspartes) | 51 (24,4%) | 32 (18,7%) |  |
| Sozialleistungen | 130 (62,2%) | 137 (80,1%) |  |
| Altersrente | 28 (13,4%) | 2 (1,2%) |  |
| Verheiratet oder in fester Partnerschaft | 170 (74,9%) | 126 (69,6%) | X²(1)=0,207; p=0,362 |
| Im Ausland geboren | 57 (25,1%) | 55 (30,4%) | X²(1)=1,41; p=0,141 |
| Psychische Erkrankungen | 49 (21,5%) | 40 (22,0%) | X²(1)=0,01; p=0,500 |
| Organische psychische Störungen | 16 (7,0%) | 4 (2,2%) | **X²(1)=5,08; p=0,019** |
| Psychotische Erkrankungen | 69 (30,1%) | 39 (21,3%) | **X²(1)=4,09; p=0,028** |
| Substanzabhängigkeit (außer Nikotin) | 69 (30,1%) | 113 (61,7%) | **X²(1)=41,23; p<0,000** |
| Schädlicher Gebrauch einer Substanz (außer Nikotin) | 34 (14,8%) | 45 (24,6%) | **X²(1)=6,23; p=0,009** |
| Affektive Störungen | 88 (38,4%) | 52 (28,4%) | **X²(1)=4,55; p=0,021** |
| Angststörungen | 9 (3,9%) | 8 (4,4%) | X²(1)=0,05; p=0,507 |
| Persönlichkeitsstörungen | 37 (16,2%) | 47 (25,7%) | **X²(1)=4,66; p=0,012** |
| Intelligenzminderungen | 10 (4,4%) | 2 (1,1%) | **X²(1)=3,86; p=0,044** |
| Anzahl psychiatrischer Diagnosen nach ICD-10 (außer Nikotin) (Median (IQR)) | 1,4 (1-2) | 1,6 (1-2) | **Z=-2,63 p=0,009** |
| Alter der ersten psychiatrischen Behandlung (Median (IQR)) | 27,0 (19,5-40,0) | 27,0 (21,0-35,0) | Z=-0,39; p=0,0698 |
| Anzahl der Teilnehmer*Innen mit einem Suizidversuch in der Vorgeschichte | 64 (27,9%) | 62 (33,9%) | X²(1)=1,69; p=0,117 |

* n=128 fehlend oder ausgeschlossen;

**1 Teilnehmer mit dem Geschlecht Transgender wurde bei Geschlecht nicht berücksichtigt;

*** Der Wohnstatus wurde in vier Gruppen eingeteilt: wohnungslose Teilnehmer*Innen (einschließlich Menschen, die direkt auf der Straße oder in sonstigen Verschlägen leben, in Notunterkünften oder sonstigen Einrichtungen der Wohnungslosenhilfe unterkommen, in Flüchtlingsheimen oder Frauenhäusern); Teilnehmer*Innen in einer eigenen Wohnung oder Wohneigentum; Teilnehmer*Innen in Einrichtungen des Gesundheitswesens bzw. der Eingliederungshilfe (therapeutischen Wohngemeinschaften, Trägerwohnungen des betreuten Einzelwohnens, Übergangswohnheime, Krankenheime etc.); Teilnehmer*Innen, die bei Freunden, Bekannten oder Familie lebten.

IQR = Interquartilsabstand
